# Phase and Frequency-Dependent Effects of Transcranial Alternating Current Stimulation on Motor Cortical Excitability

**DOI:** 10.1371/journal.pone.0162521

**Published:** 2016-09-08

**Authors:** Hisato Nakazono, Katsuya Ogata, Tsuyoshi Kuroda, Shozo Tobimatsu

**Affiliations:** 1 Department of Clinical Neurophysiology, Neurological Institute, Faculty of Medicine, Graduate School of Medical Sciences, Kyushu University, Fukuoka, Japan; 2 Faculty of Informatics, Shizuoka University, Shizuoka, Japan; University Medical Center Goettingen, GERMANY

## Abstract

Transcranial alternating current stimulation (tACS) can entrain ongoing brain oscillations and modulate the motor system in a frequency-dependent manner. Recent animal studies have demonstrated that the phase of a sinusoidal current also has an important role in modulation of neuronal activity. However, the phase effects of tACS on the human motor system are largely unknown. Here, we systematically investigated the effects of tACS phase and frequency on the primary motor cortex (M1) by using motor evoked potentials (MEPs) with transcranial magnetic stimulation (TMS). First, we compared the phase effects (90°, 180°, 270° or 360°) of 10 and 20 Hz tACS on MEPs. The 20 Hz tACS significantly increased M1 excitability compared with the 10 Hz tACS at 90° phase only. Second, we studied the 90° phase effect on MEPs at different tACS frequencies (5, 10, 20 or 40 Hz). The 20 vs. 10 Hz difference was again observed, but the 90° phase in 5 and 40 Hz tACS did not influence M1 excitability. Third, the 90° phase effects of 10 and 20 Hz tACS were compared with sham stimulation. The 90° phase of 20 Hz tACS enhanced MEP amplitudes compared with sham stimulation, but there was no significant effect of 10 Hz tACS. Taken together, we assume that the differential 90° phase effects on 20 Hz and 10 Hz tACS can be attributed to the neural synchronization modulated by tACS. Our results further underline that phase and frequency are the important factors in the effects of tACS on M1 excitability.

## Introduction

Rhythmic brain activity is generated by neuronal elements or networks of different spatial scales [1]. Regional oscillatory activities of specific frequencies are related to distinct brain functions [2]. For instance, spontaneous oscillations such as α (8–13 Hz) and β (14–30 Hz) frequency bands are observed in the sensorimotor area. These oscillations are desynchronized during execution or imagination of movement [3–6]. Conversely, corticomuscular synchronization has been shown in the α and β range during isometric muscle contraction [7–9] and slow finger movement [10], respectively. These findings suggest that brain oscillations (i.e., α and β bands) play an important role in the modulation of the human motor system. However, the precise functions of neural oscillations in the sensorimotor area are largely unknown.

Recently, transcranial alternating current stimulation (tACS) has been applied to manipulate neural oscillations in certain brain areas, and a causal link between brain oscillations and distinct functions has been elucidated in these studies [11–14]. tACS modulates ongoing oscillatory activity in a frequency-dependent manner [15–17], and the effective stimulation frequency is regarded as matching the targeted brain oscillations [18–20]. Previous studies have examined how motor cortex excitability or motor performance is modulated by tACS in the α and β ranges (i.e., 10 and 20 Hz) [21–30]. For instance, 20 Hz tACS increased the excitability of the primary motor cortex (M1) measured by motor evoked potentials (MEPs) [21–23], while 10 Hz tACS did not show such a significant effect on MEPs [21,22,24,25]. Regarding the behavioral effects, 20 Hz tACS slowed voluntary movements [26–28], whereas 10 Hz tACS increased movement variability, especially in tasks requiring pacing [26]. Similarly, motor sequence learning was differentially affected by 10 and 20 Hz tACS, depending on the stimulation state (during or after stimulation) [29,30]. These results suggest that 10 and 20 Hz tACS have differential effects on sensorimotor areas by modulating ongoing α and β oscillations.

In addition to the stimulus frequency, the phase of the sinusoidal current used in tACS is also a crucial factor in the modification of neuronal activity or perceptual performance. In animal studies, the physiological effects of tACS have been studied via intracranial recordings [31,32]. Interestingly, these studies showed that tACS synchronized neuronal spiking to the phase of the sinusoidal current, i.e., tACS could modulate the timing of neural activity in a phase-specific manner. Similarly, spontaneous brain oscillations were also entrained by applying tACS, which modulated the target detection performance in a phase-dependent fashion in human auditory or visual processing [11,16,33]. However, it is unclear whether tACS phase influences cortical neural excitability, and there is no evidence supporting phase effects of 10 and 20 Hz tACS on M1. According to the results of animal studies, it is likely that tACS modulates ongoing oscillations as well as neuronal activity in the human sensorimotor area. This then predicts the modification of MEP amplitudes, depending on the phase of 10 and 20 Hz tACS.

To solve these issues, the present study was conducted to examine whether the effects of tACS frequency on M1 excitability depend on the phase of its sinusoidal current. More specifically, we manipulated not only the frequency of tACS but also the phase at which single-pulse transcranial magnetic stimulation (TMS) was applied to record the MEPs. To achieve this, we performed three experiments. In Experiment 1, both the frequency (10 and 20 Hz) and the phase (90°, 180°, 270°, and 360°) of tACS were manipulated. In Experiment 2, more frequencies (5, 10, 20 and 40 Hz) were adopted but the phase was fixed at 90°, which was found to be the most effective in Experiment 1. In Experiment 3, the 90° phase effects of 10 and 20 Hz tACS were compared with sham conditions.

## Materials and Methods

### Participants

A total of 38 healthy volunteers (15 female, mean age ± SD: 25.2 ± 5.5 years) participated in three experiments, and were recruited from August 2014 to July 2016. None of the participants had any history of neurological, psychiatric, or other medical problems. All participants were right-handed, according to the Edinburgh handedness inventory [34]. Written informed consent was obtained from each participant in accordance with the Declaration of Helsinki. The experimental protocol was approved by the Ethics Committee of Kyushu University.

### tACS

Participants were seated on a comfortable chair and instructed to keep their muscles relaxed during the experiment. tACS was delivered by a battery-driven current stimulator (DC Stimulator-Plus, NeuroConn GmbH, Ilmenau, Germany) through two self-adhesive electrodes (PALS electrodes, Axelgaard Manufacturing Co., Ltd., Fallbrook, CA, USA). tACS without DC offset was applied at 1000 μA (peak-to-peak), and the maximal current density was 28.6 μA/cm^2^. The tACS electrode (5 × 7 cm) was placed over the “hot spot” of the left M1 as determined by TMS, whereas the other electrode was placed on the midline parietal region (Pz; International 10–20 system). The positions of the stimulation electrode were adopted according to previous studies [21,22] that demonstrated frequency- and region- specific tACS effects on M1 excitability, and to avoid the flickering sensation on the retina that could be induced by volume conduction from the electrode [35]. Scalp skin around left M1 and Pz was cleaned with alcohol, and the electrode gel (Gelaid, Nihon Kohden, Tokyo, Japan) was applied to reduce the electrode impedance. The impedance was kept below 5 kΩ. The electrodes were fixed using a support bandage. The current was ramped up and down for 2 s to reduce skin sensation.

### MEPs

Single-pulse TMS was delivered over the hand area of the left M1 using a 70 mm figure-of-eight coil connected with a monophasic Magstim 200 stimulator (Magstim Co., Whitland, UK). MEPs were recorded from the right first dorsal interosseous muscle (FDI). The coil was held tangential to the scalp with the handle pointing posterolaterally at about 45° from the midline. The hot spot for FDI was determined as the site where TMS of slightly suprathreshold intensity consistently elicited the largest MEP amplitudes. The position was marked with a pen for repositioning the coil. TMS was applied over the tACS electrode overlying the left M1, and the TMS intensity was adjusted to elicit an MEP amplitude of 0.5–1.0 mV in the relaxed muscle. The intensity was kept constant throughout the experiments. TMS was applied every 7 s. Muscle relaxation was maintained online via visual feedback of electromyographic (EMG) activity.

Compound muscle action potentials of the FDI were recorded in a belly-tendon derivation using Ag/AgCl electrodes. Signals were amplified (Neuropack 8, Nihon Kohden, Tokyo, Japan) with a band-pass filter of 10 Hz–2 kHz, digitized at a sampling rate of 10 kHz and stored in a computer using signal processing software (Multiscope PSTH, Medical Try System, Tokyo, Japan) for offline analysis. The analysis period was 500 ms in length, beginning 250 ms before TMS.

TMS and tACS were controlled by Presentation software (Neurobehavioral Systems, Albany, CA, USA). The timing of the TMS pulse to the targeted tACS phase was calculated from the time difference. For instance, if the TMS pulse was synchronized with the phase of 10 Hz tACS, the 90° phase difference was calculated as a 25 ms time difference. The relationship between tACS phase and TMS pulse was monitored online. Therefore, this study was a single-blind design.

### Procedures

#### Experiment 1: Phase effects of 10 and 20 Hz tACS

**Fig 1:**
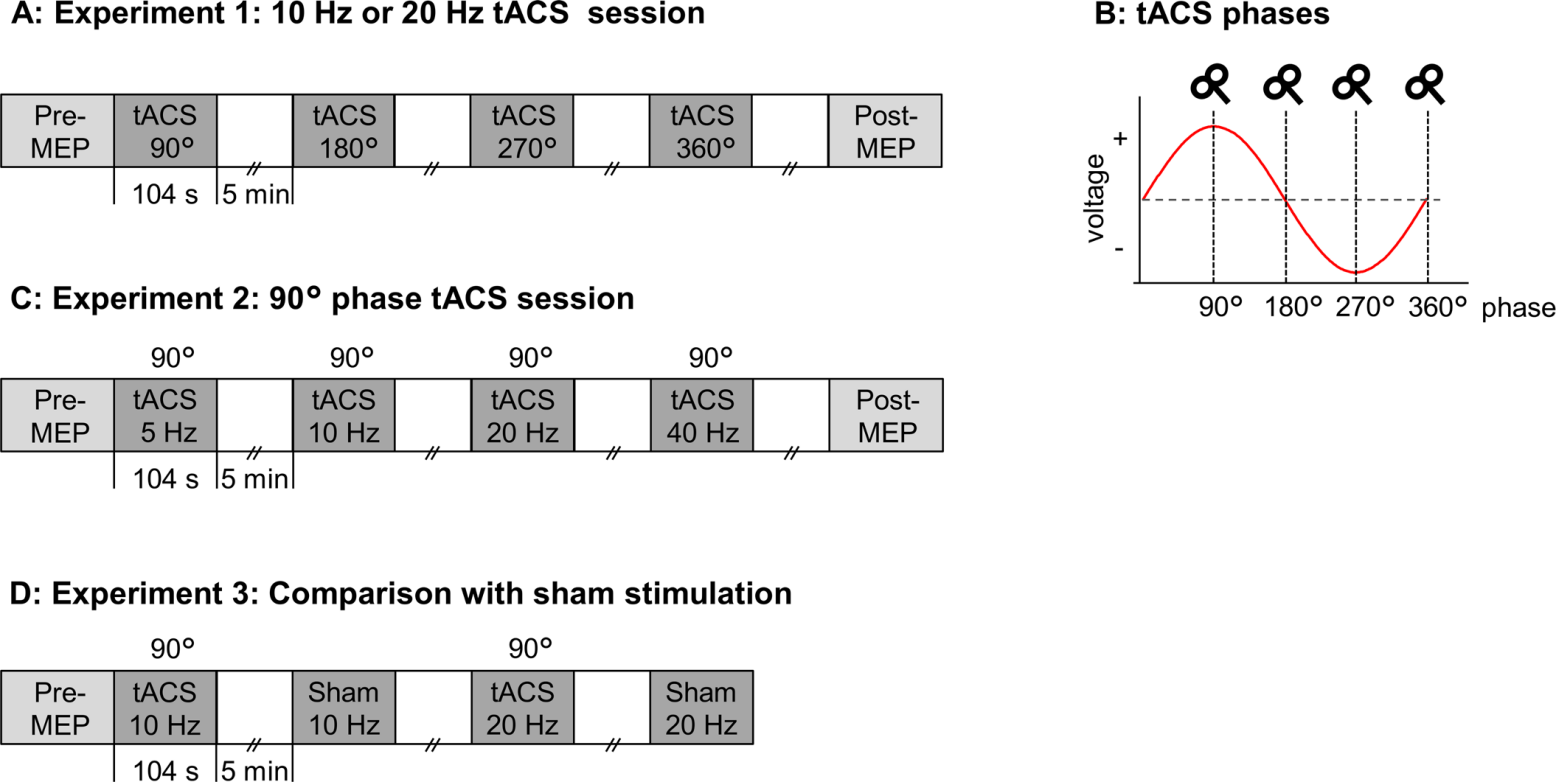
Experimental procedures. (A) Time course of 10 and 20 Hz tACS sessions in Experiment 1. MEPs were recorded online at different tACS phases. MEPs were also recorded at pre- and post-tACS trials. (B) The four phases of tACS (90°, 180°, 270° or 360°) for TMS in Experiment 1. (C) Time course of Experiment 2. tACS at different frequencies (5, 10, 20, or 40 Hz) was applied online, while the TMS timing was fixed at 90° phase in each frequency. (D) Time course of Experiment 3. Two real tACS (10 and 20 Hz tACS) and two sham stimulations (10 and 20 Hz tACS) were carried out. For the real conditions, the TMS timing was fixed at 90° phase for each frequency.

Sixteen participants (6 females, 26.1 ± 5.5 years) took part in a crossover design study (i.e., 10 and 20 Hz tACS sessions). These two sessions were separated by at least 2 days, and the order of frequency was counterbalanced among the participants. The time course of Experiment 1 is shown in Fig 1A. Each session consisted of four online tACS trials. The timing of TMS was adjusted to one of the tACS phases (90°, 180°, 270° or 360°) in each trial (Fig 1B). The order of the tACS trials (90°, 180°, 270° or 360°) was counterbalanced among the participants. TMS was started 20 s after the beginning of tACS, and 12 MEPs were recorded [21,22]. tACS was delivered for 104 s in each trial, and the inter-trial interval (ITI) was 5 min. We also obtained 12 MEPs in pre- and post-online trials to evaluate the stability of baseline MEP amplitudes and the after-effects of short-lasting tACS.

#### Experiment 2: Frequency-dependent phase effects

Fifteen participants (6 female, 26.3 ± 5.3 years) took part in this experiment (10 participants were the same as those who participated in Experiment 1). We performed four online trials to elucidate the 90° phase effect at different stimulation frequencies (Fig 1C). Four tACS frequencies (5, 10, 20 or 40 Hz) were used, and the timing of TMS was adjusted to 90° phase for all stimulation frequencies. The order of the four trials for tACS frequencies was counterbalanced among the participants. The other procedures were identical to those of Experiment 1.

#### Experiment 3: Comparison with sham conditions

Seventeen participants (7 female, 24.2 ± 5.7 years) took part in this experiment, all of whom were recruited separately from those of Experiments 1 and 2. We performed four online trials that consisted of two real stimulation (10 and 20 Hz tACS) and two sham stimulation conditions (10 and 20 Hz tACS) (Fig 1D). The order of the four trials for tACS conditions was counterbalanced among the participants. In the real conditions, the 90° phase effects of 10 and 20 Hz tACS were evaluated by TMS similar to Experiments 1 and 2. For the sham conditions, the stimulation characteristics (i.e., stimulation frequency and the timing of TMS) were the same as in the real conditions, but tACS was applied only for the first 10 s. In Experiments 1 and 2, a few participants perceived a slight flickering sensation during the 20 Hz tACS conditions (see results). Therefore, we performed two sham conditions (i.e., 10 and 20 Hz tACS) to evaluate the possibility of whether tACS-induced perception influenced the tACS effects. To achieve this, the 20 Hz real and 20 Hz sham (or 10 Hz real and 10 Hz sham) stimulation conditions were compared. We omitted to obtain MEPs in post-online trials because we observed no cumulative effects in the short tACS sessions in Experiments 1 and 2.

### Data Analysis

Peak-to-peak amplitudes of MEPs were measured and then log-transformed. They were subsequently averaged for each trial. Raw data are provided as S1 Dataset. This log transformation was used to stabilize variances across the trials and to reduce outlier effects [36].

To evaluate the effects of the phase and frequency on MEP amplitudes, two-way repeated measures analysis of variance (ANOVA) was employed with factors of phase (90°, 180°, 270° and 360°) and frequency (10 and 20 Hz) for the first experiment. For Experiment 2, one-way ANOVA was conducted with the factor of frequency (5, 10, 20 and 40 Hz). In Experiment 3, two-way ANOVA was performed with factors of condition (tACS and sham) and frequency (10 and 20 Hz). The Greenhouse-Geisser correction was used when sphericity was lacking. A post-hoc analysis was performed using paired *t*-tests with Bonferroni’s correction for multiple comparisons. To test MEPs’ stability for pre- and post-online trials (offline trials), two-way ANOVA was applied with factors of time (pre and post) and frequency (10 and 20 Hz) in Experiment 1, and paired *t*-tests were applied to the offline trials in Experiment 2 (pre vs. post). Three control conditions (pre and 10 and 20 Hz sham tACS) in Experiment 3 were also analyzed by one-way ANOVA. The data are presented as means ± standard error of the mean. Statistical analysis was carried out with SPSS (version 17.0 for Windows, IBM, Armonk, NY, USA).

## Results

### Experiment 1: Phase Effects of 10 and 20 Hz tACS

As observed in previous studies [37,38], two participants felt a slight itching sensation, but they were unable to recognize the difference between the tACS conditions. Three participants reported a slight flickering sensation in their peripheral visual fields during 20 Hz tACS. These skin itch and flickering sensations were perceived at the beginning of the stimulation and then faded away.

**Fig 2:**
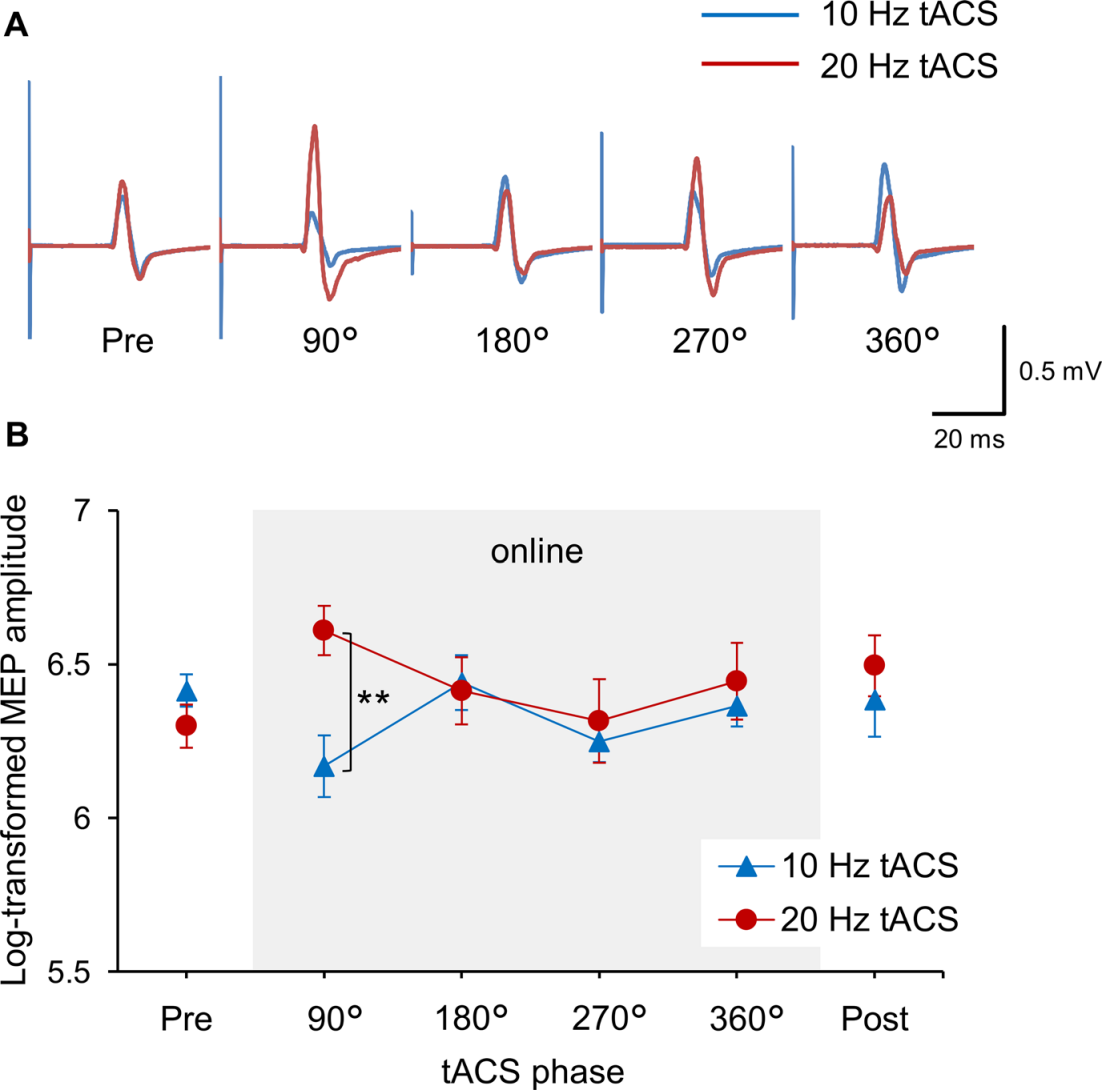
Phase effects of 10 and 20 Hz tACS. (A) Representative examples of MEPs from before and online trials (phase effects) of 10 and 20 Hz tACS. The blue lines represent MEPs from a 10 Hz tACS session while the red lines represent MEPs from a 20 Hz tACS session. (B) Mean MEP amplitudes before, online and after stimulation in 10 and 20 Hz sessions. The gray-shaded area shows online effects of tACS at different phases (90°, 180°, 270° or 360°). There was a significant difference between the tACS sessions at 90° phase only, indicating the opposite effects of the two frequencies. Error bars indicate standard error of the mean. ** *p* < 0.01.

There were no significant differences in TMS intensity (10 Hz, 59.4 ± 2.3%; 20 Hz, 60.3 ± 2.5% of maximum stimulator output) or baseline MEP amplitudes (10 Hz, 749.5 ± 37.4 μV; 20 Hz, 728.7 ± 45.8 μV) between the experimental sessions (*p* > 0.201). Fig 2A shows MEP waveforms recorded from a representative participant before and during 10 and 20 Hz tACS stimulation. TMS was given to the participants when the sinusoidal current reached 90°, 180°, 270° or 360° phase. The MEP amplitudes tended to increase for 20 Hz tACS but to decrease for 10 Hz tACS when they were recorded at 90° phase. However, this 10 vs. 20 Hz difference was not found for the other phases.

Fig 2B shows the mean log-transformed MEP amplitudes for the 10 and 20 Hz tACS sessions. Two-way ANOVA revealed a significant interaction between frequency and phase (*F*(3, 45) = 2.902, *p* = 0.045) without significant main effects of frequency (*F*(1, 15) = 2.699, *p* = 0.121) or phase (*F*(3, 45) = 1.377, *p* = 0.262). The post-hoc analysis showed that 20 Hz tACS significantly increased M1 excitability compared with 10 Hz tACS at only 90° phase (90° phase; *p* = 0.002, other phases; *p* > 0.603). Focusing on individual responses, 13 of the 16 participants exhibited a higher MEP amplitude for 20 Hz tACS than for the 10 Hz condition at 90° phase.

For the offline trials, neither significant effects of time (*F*(1, 15) = 0.743, *p* = 0.402), frequency (*F*(1, 15) = 0.001, *p* = 0.98), nor significant interaction between frequency and time (*F*(1, 15) = 2.453, *p* = 0.138) were observed, which indicated that there were no cumulative effects in the short tACS sessions with 5 min intervals for both frequencies.

### Experiment 2: Frequency-Dependent Phase Effects

Two participants felt a slight itching sensation at all frequencies, and two participants saw a slight flickering in their peripheral visual fields during 20 Hz tACS. These sensations were perceived at the beginning of the stimulation and then faded away.

**Fig 3:**
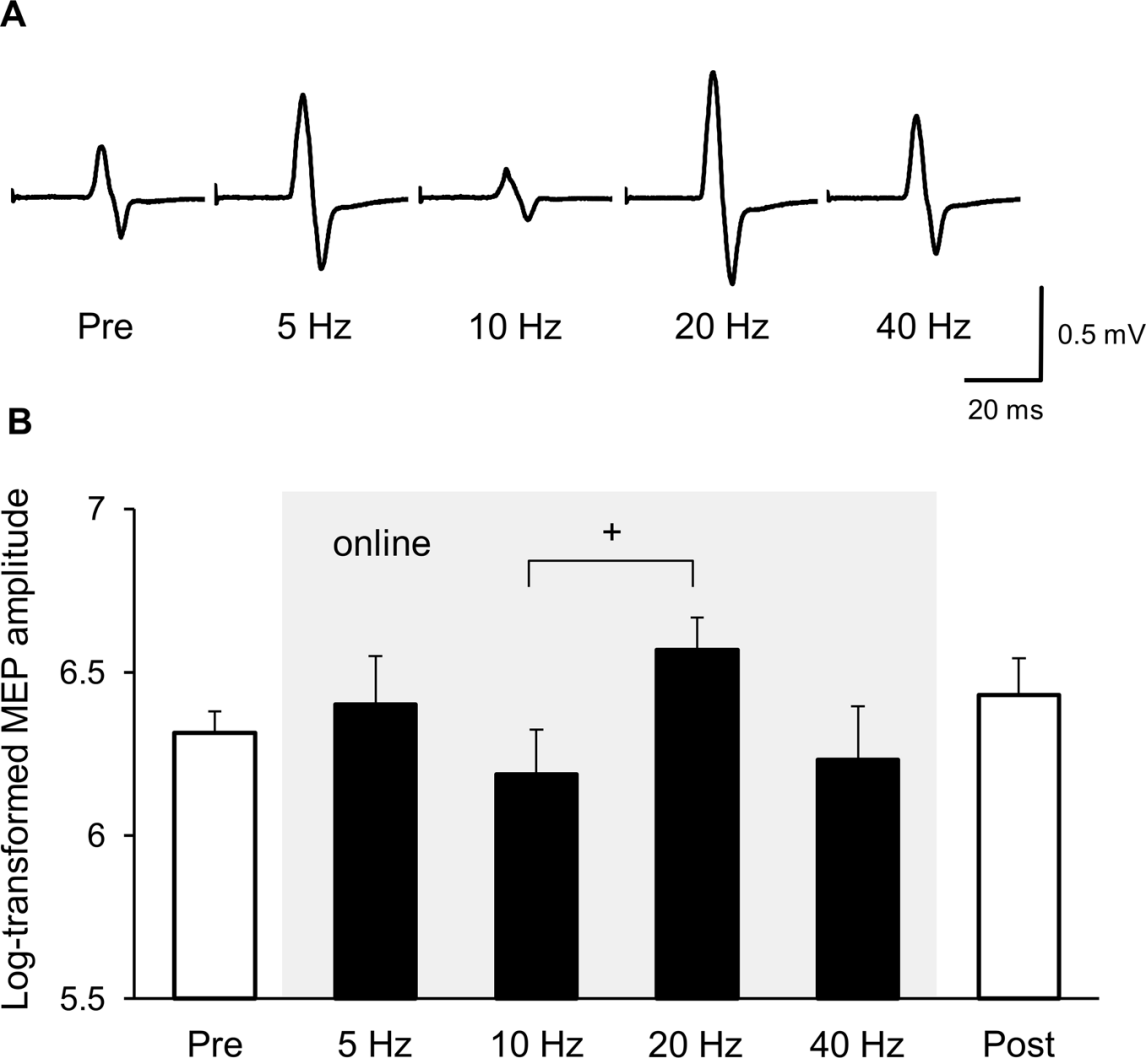
Frequency-dependent phase effects of tACS. (A) Representative examples of MEPs from before and online trials (frequency effects at 90° phase) of tACS. (B) Mean MEP amplitude before, online and after tACS. The gray-shaded area shows online effects of tACS with different frequencies (5, 10, 20, or 40 Hz) at 90° phase. There was a marginally significant difference between 10 and 20 Hz tACS (+ *p* < 0.1), but all other pairwise comparisons revealed no significant differences (*p* > 0.1).

The mean TMS intensity was 57.4 ± 2.2% of the maximum stimulator output, and the mean baseline MEP amplitude was 704.1 ± 39.3 μV. Fig 3A shows MEP waveforms recorded from a representative participant before and during tACS with different frequencies (5, 10, 20 or 40 Hz) at 90° phase. Similar to Experiment 1, a clear difference between 10 and 20 Hz tACS was noted.

Fig 3B shows the mean log-transformed MEP amplitudes from before, online (frequency effects at 90° phase), and after tACS. One-way ANOVA revealed a significant main effect of frequency (*F*(3, 42) = 3.087, *p* = 0.037). In the post-hoc analysis, 20 Hz tACS showed a tendency toward higher MEP amplitude compared with 10 Hz tACS (*p* = 0.067). Indeed, 12 of the 15 participants showed higher MEP amplitudes for 20 Hz tACS compared with the 10 Hz condition, thus replicating almost the same 10 vs. 20 Hz differences as found in Experiment 1 (13 of 16 participants). However, none of the other pairwise comparisons showed significant differences (*p* > 0.164). For the offline trials, there were no significant differences between the pre- and post-online trials (*p* > 0.32).

### Experiment 3: Comparison with Sham Conditions

Two participants felt a slight itching sensation in all conditions, including sham conditions. One participant perceived a slight flicker in peripheral visual fields during 20 Hz real and sham tACS conditions, but was unable to discriminate the difference between the two conditions.

**Fig 4:**
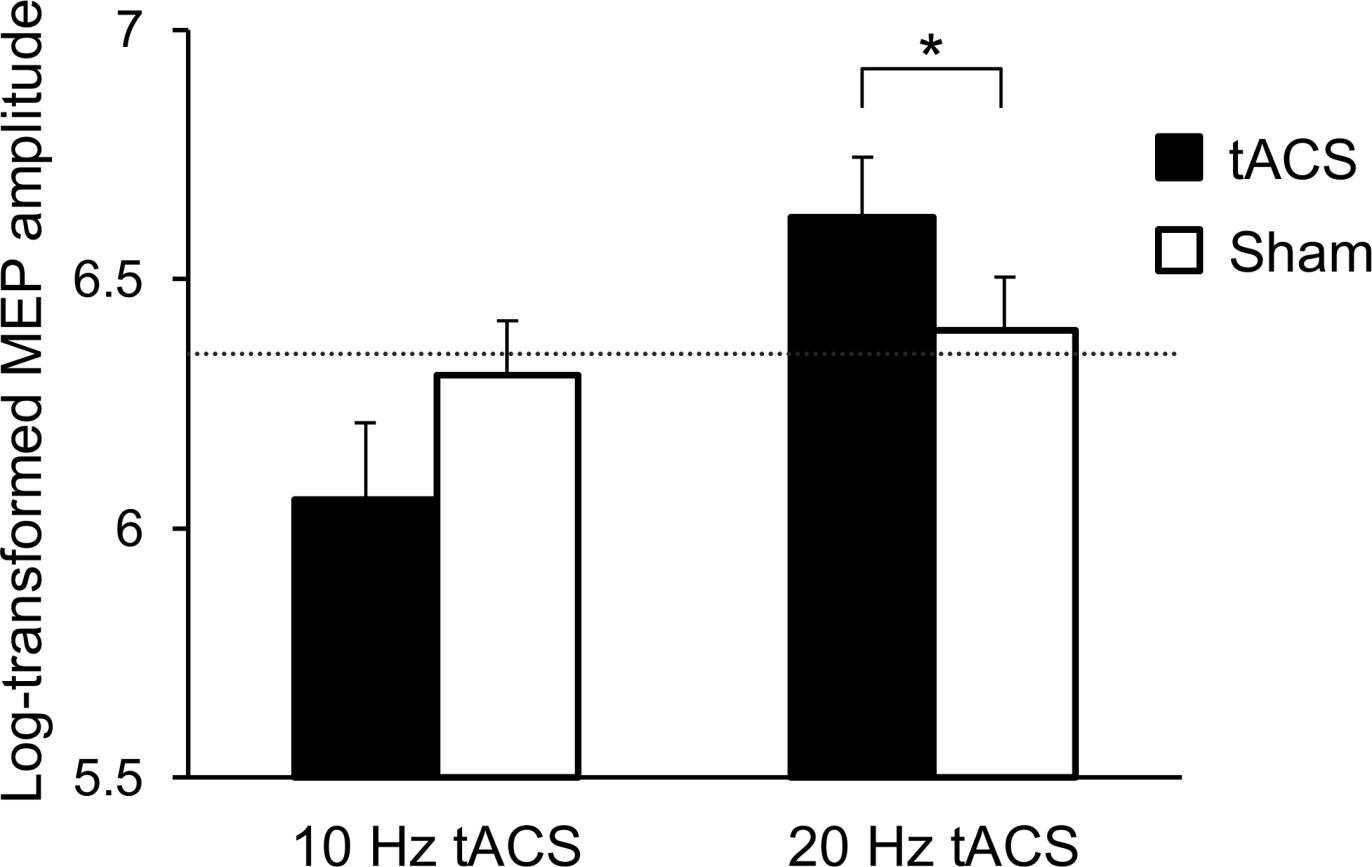
Comparison with sham stimulation. Bar graphs show the mean MEP amplitudes of the real tACS conditions (90° phase of 10 and 20 Hz tACS) and those of the sham conditions (10 and 20 Hz tACS). A dashed line indicates the mean baseline MEP amplitudes. There was a significant difference between the 20 Hz tACS at 90° phase and sham condition of 20 Hz tACS, while the 10 Hz tACS did not show a significant change. * *p* < 0.05.

The mean TMS intensity was 60.5 ± 2.5% of the maximum stimulator output, and the mean baseline MEP amplitude was 775.8 ± 31.1 μV. Fig 4 shows the mean log-transformed MEP amplitudes from the real and sham stimulations. Two-way ANOVA revealed a significant effect of frequency (*F*(1, 16) = 7.566, *p* = 0.014) and a significant interaction between frequency and condition (*F*(1, 16) = 7.712, *p* = 0.013). However, a significant main effect of condition was not observed (*F*(1, 16) = 0.01, *p* = 0.924). The 90° phase of 20 Hz tACS significantly increased MEP amplitudes compared with sham stimulation of 20 Hz tACS (*p* = 0.03), while 10 Hz tACS did not show a significant change (*p* = 0.185). For the control conditions, one-way ANOVA revealed no significant difference among the pre- and two sham conditions (*F*(2, 32) = 0.228, *p* = 0.797).

## Discussion

Our aim was to elucidate the effects of tACS phase and frequency on M1. In Experiment 1, 20 Hz tACS increased MEP amplitudes compared with those associated with 10 Hz tACS at 90° phase only. This 90° phase effect was not observed at the other frequencies (5 and 40 Hz). Moreover, 90° phase at 20 Hz but not 10 Hz tACS enhanced M1 excitability compared with sham stimulation. Taken together, our results clearly demonstrated that not only frequency but also tACS phase is important for the effects of tACS on M1 excitability.

### Phase and Frequency Dependency of tACS Effects

The underlying mechanism of the tACS effect has not been fully elucidated. However, there is growing evidence that tACS entrains neural oscillations in animals and humans [11,12,16,17,21,22,31,32,39]. Regarding the frequency-dependent effects of tACS over M1, Feurra et al. [21,22] reported that 20 Hz tACS increased MEP amplitudes during stimulation compared with other frequencies (5, 10 and 40 Hz). They assumed that 20 Hz tACS entrained endogenous β oscillations of the resting M1. In the current study, although 20 Hz tACS over M1 increased MEP amplitudes compared with 10 Hz tACS, this effect was dependent on the 90° phase of tACS in Experiment 1. Moreover, the 90° phase of 20 Hz tACS enhanced MEP amplitudes compared with sham stimulation in Experiment 3. Hence, our findings demonstrated that 20 Hz tACS facilitates M1 excitability in a phase-dependent manner, which extends the results of Feurra et al. [21,22] and supports the proposition that 20 Hz tACS entrains β oscillations in M1.

In contrast to the 20 Hz tACS effects, 10 Hz tACS tended to decrease MEP amplitudes at 90° phase in all experiments, but there was no significant difference when compared with sham stimulation. Although previous studies revealed that 10 Hz tACS did not change M1 excitability during or after stimulation [21,22,25], our results showed a difference in 90° phase effects between 10 and 20 Hz tACS in Experiment 2. This implies that 10 Hz tACS may have a weak frequency-dependent effect on the resting M1. Indeed, Antal et al. [24] reported that 10 Hz tACS tends to decrease MEP amplitudes. Moreover, several studies have shown that 10 Hz tACS over M1 modulates motor behavior [26] or motor learning [24,29], and these effects are different from the modulation of 20 Hz tACS [26,29,30].

Regarding the causal interaction between tACS and ongoing brain oscillations, recent human studies have suggested that tACS modulates endogenous oscillatory activity, as indicated by electroencephalography (EEG) when the stimulation frequency matches the targeted brain oscillations [11,15–17,40,41]. For instance, several studies have shown that tACS at α range increases α oscillatory activity in the occipital cortex [15,40,41]. Helfrich et al. [16] successfully demonstrated that 10 Hz tACS increased parieto-occipital α activity with simultaneous tACS-EEG recordings. Moreover, the manipulation of the phase of tACS resulted in improvement of the detection performance in human auditory [11,33] and visual [16] modalities in a phase-dependent manner. These previous results have been interpreted as showing that tACS entrains the cortical oscillations related to perception. In line with those studies, the phase effects of 10 and 20 Hz tACS may reflect the relationship between ongoing oscillations (i.e., α and β ranges) and M1 excitability. In combined TMS-EEG studies, though a link between the ongoing oscillations prior to TMS and MEP amplitudes was not fully established [42–48], Keil et al. [49] reported that MEP amplitudes increased depending on the phase of β oscillations. Their results would partly support the phase effect of 20 Hz tACS in our study. Moreover, the source of rolandic β rhythms has been shown to originate from M1, while α rhythms have been attributed to the somatosensory system [3,5,50]. Therefore, we assume that the effects of 20 Hz tACS are more apparent than those of 10 Hz, because 20 Hz tACS entrains β oscillations that are inherent oscillations in M1. Thus, future studies are needed to evaluate endogenous oscillations during TMS and tACS to address this hypothesis.

### Physiological Effects of tACS Phase

In terms of the physiological mechanisms of tACS, recent animal studies have evaluated the neuronal spiking activities via extracellular recordings during externally applied alternating current (AC) [31,32,39,51]. The results indicate that AC stimulation could entrain the neural firings to different driving frequencies [51] and synchronize the spiking activity to the AC phase [31,32,39]. Modeling studies have also suggested that positive-phase AC stimulation could synchronize the neural firings [32,51]. Furthermore, AC stimulation entrains firing activity when the stimulation frequency is matched with the endogenous rhythm frequency [39,52]. In the motor cortex, oscillations in the β range have been observed in local field potential recordings of monkeys [53]. Several studies have suggested that cortical neurons in M1 tend to fire at β range, and might play a role in the generation of β oscillatory activity [54–56]. In line with these findings, 20 Hz tACS may synchronize the neural firings at 90° phase, resulting in altered M1 excitability.

Meanwhile, it has been assumed that stronger α activity in the sensorimotor area reflects a state of functional inhibition [57,58]. Interestingly, Haegens et al. [59] reported that increased α power in the sensorimotor area was associated with a decrease in M1 neuron firing rate in animal models. They also showed that the firing rate of M1 was the lowest at 90° phase of α oscillations [59]. Therefore, it is conceivable that M1 neuronal activities are decreased at 90° phase of 10 Hz tACS, which would be compatible with our results.

### Limitations

We acknowledge several limitations of our study. First, the phase effects of tACS in the theta (5 Hz) and low-gamma (40 Hz) range were not examined systematically in this study. Future studies need to clarify the phase effects of tACS at these frequencies. Second, a double-blind designed study was not performed because we needed to monitor the precise timing between TMS pulses and tACS phase. Further studies will be double-blind if possible. Although a few participants perceived a slight flickering sensation only in the 20 Hz tACS conditions, these perceptions cannot explain the phase effects of 10 and 20 Hz tACS because most participants were unable to discriminate between the different tACS frequency conditions. Moreover, we observed a significant difference between 20 Hz tACS real and sham conditions in Experiment 3, under which no participant could recognize the difference between real and sham conditions. Finally, we used rectangular stimulation electrodes with a size of 5 × 7 cm (35 cm^2^); such large electrodes did not allow for a very focal stimulation, as used in modeling studies [60,61]. Thus, it is difficult to rule out the co-stimulation of cortical areas adjacent to M1.

## Conclusions

We found an important relationship between tACS phase and frequency (10 and 20 Hz) during stimulation over M1. Our results suggest that tACS modulates neural activity in phase- and frequency-dependent manners. Hence, the selection of stimulus parameters such as phase and frequency are extremely important for tACS experiments on M1 excitability. This finding may contribute to the therapeutic application of tACS in the future.

## Supporting Information

S1 Dataset The data for MEP amplitudes and TMS intensity. (XLSX)
